# Supplementary material for: Systematic proteome and proteostasis profiling in human Trisomy 21 fibroblast cells
Source: Nat Commun. 2017 Oct 31;8:1212. doi: 10.1038/s41467-017-01422-6 (PMC5663699; doi:10.1038/s41467-017-01422-6)
Supplement: Supplementary file 1 — Supplementary Information [file 41467_2017_1422_MOESM1_ESM.pdf]

Supplementary Figures

Supplementary Figure 1.

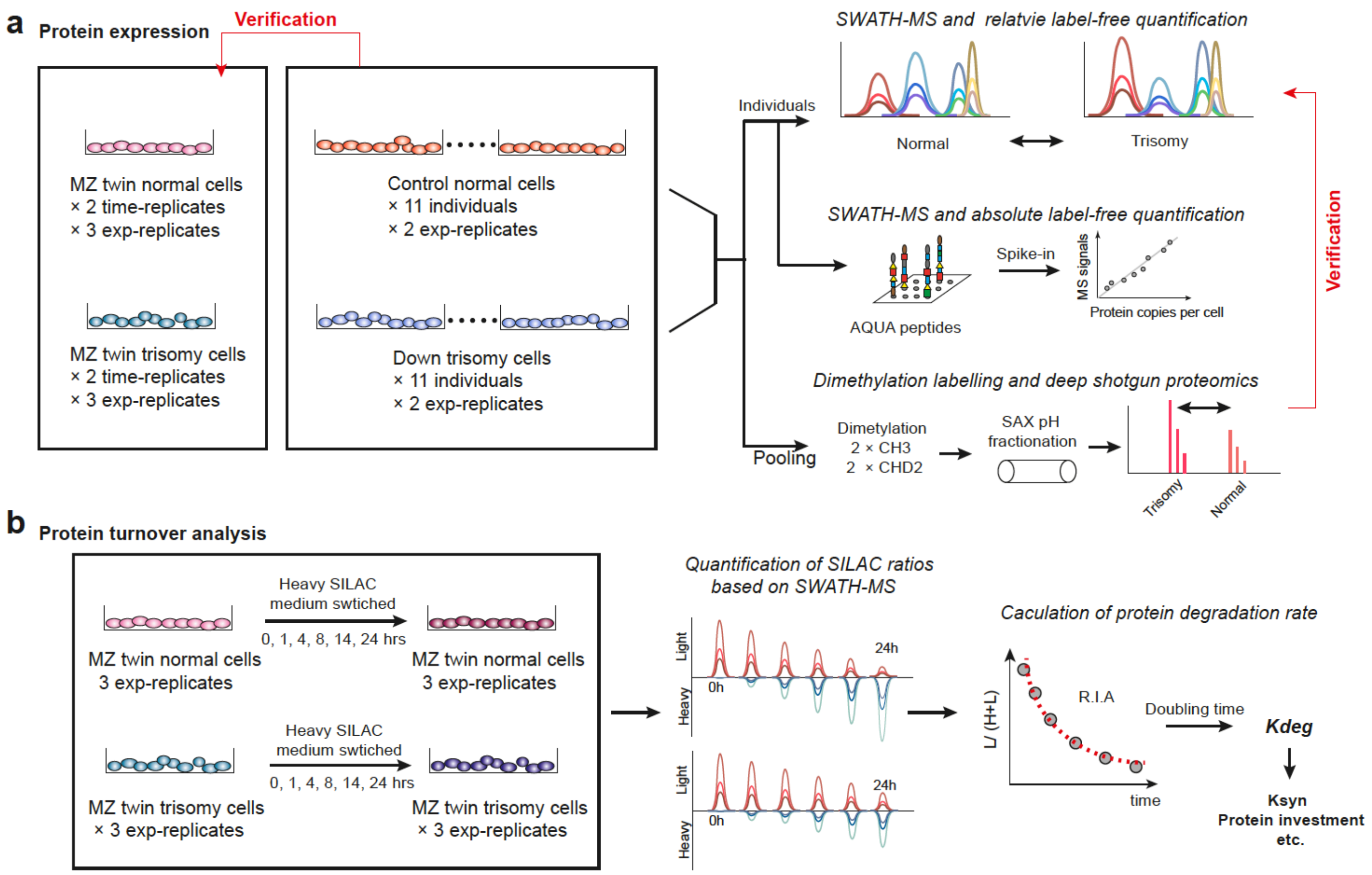

Supplementary Figure 1 The entire experimental design for protein expression and turnover analysis. (a) For the steady state protein expression measurement, we used SWATH-MS to analyze the proteome of fetal skin primary fibroblasts derived from a pair of MZ twins discordant for T21 ("twin samples" of T2N and T1DS, where N denoting normal and DS denoting Down Syndrome) and 11 DS and 11 unrelated non-DS individuals ("Unrelated samples"). For each twin two independent cell cultures (cultured in two times with 1.5 months in between) and three experimental replicates for each were analyzed, resulting in six proteomic profiles per individual. For each unrelated sample, two experimental replicates were performed. The "experimental replicate" essentially means the independent whole-process replicate starting from two separated dishes (at one time point) of cells to the final mass spectrometry data acquired. **(b)** For the pulsed SILAC (pSILAC) analysis, the experiments and data analysis were performed as described to twin samples. We used an alternative Dimethylation labeling method to confirm SWATH-MS results (See Figure S2).

Supplementary Figure 2.

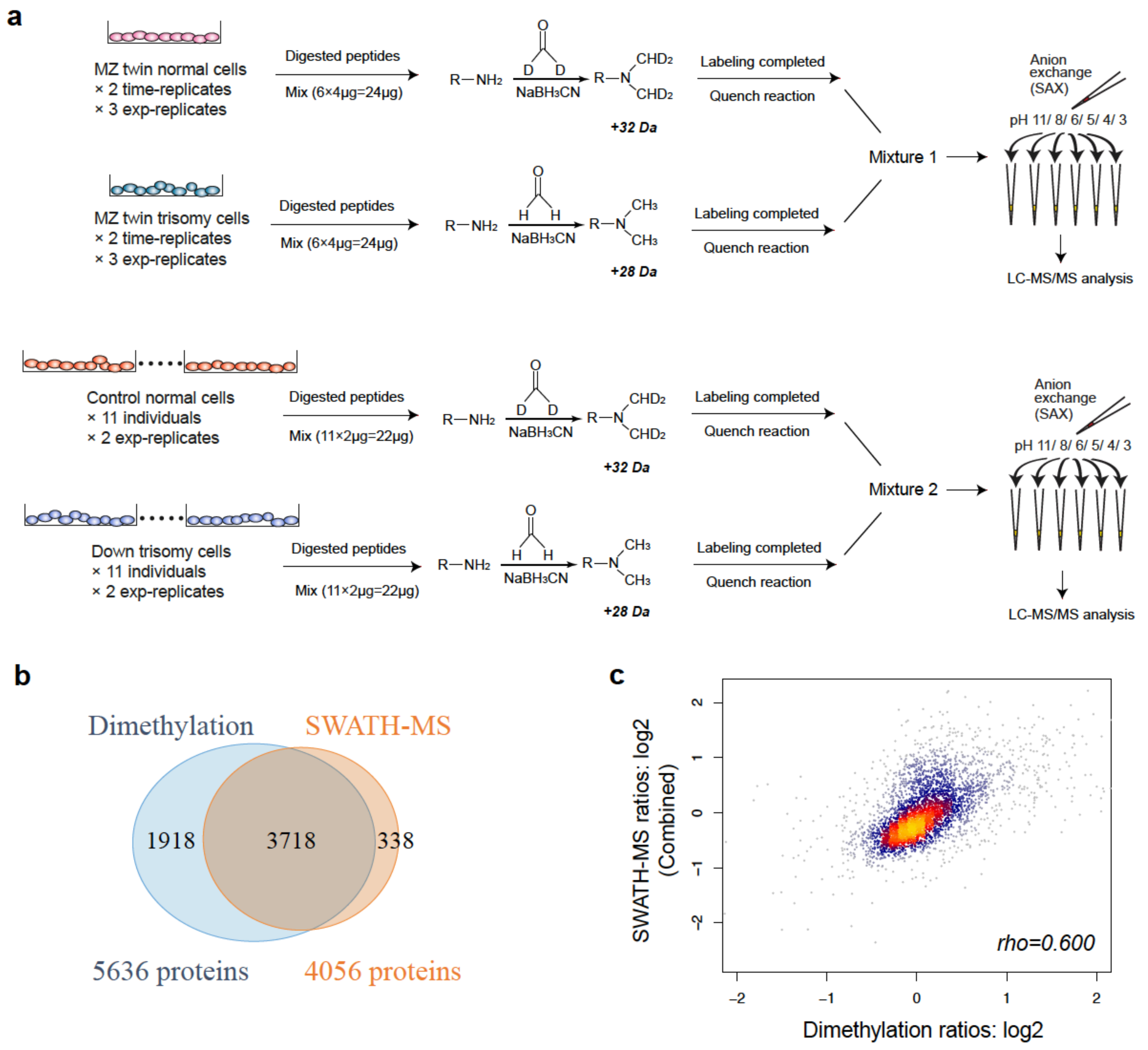

Supplementary Figure 2 The Dimethylation labelling proteomics as an alternative quantification method confirming SWATH-MS results. (a) Detailed experimental schema. This method combined (1) dimethyl labeling of mixed T21 samples and Normal samples from the respective "twin" and "unrelated" samples, (2) the SAX fractionation of the peptide digested and labeled from each sample, (3) shotgun proteomic analysis as described the Methods and Materials. **(b)** Venn diagram of identifications from Dimethyl labeling shotgun proteomics (15 hours per sample) and SWATH-MS (2.5 hours per sample). **(c)** The quantitative correlation between log2 FC of T1DS/T2N for proteins identified by both methods, yielding a Spearman correlation of rho=0.6.

Supplementary Figure 3.

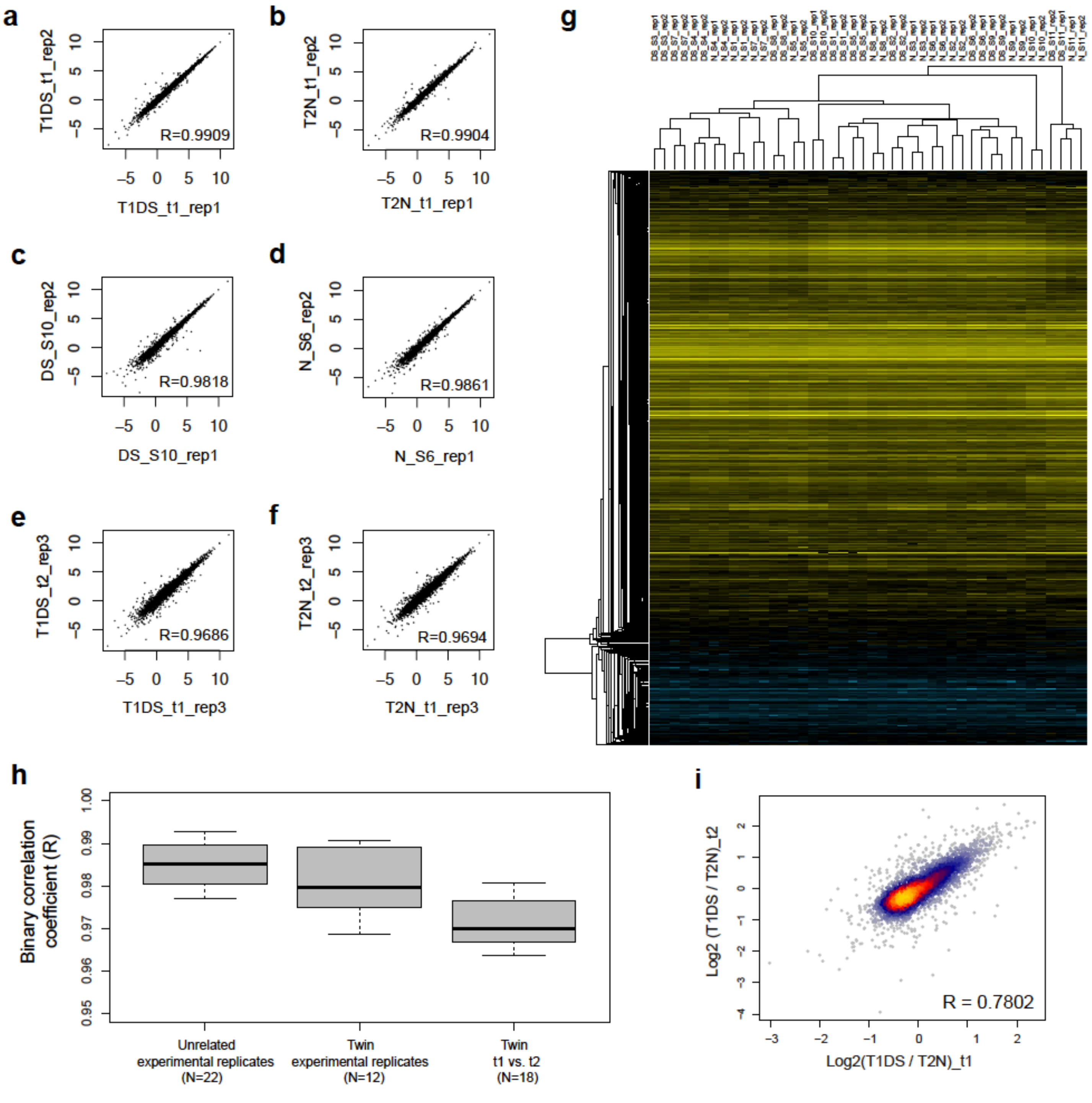

Supplementary Figure 3 Experimental and technical reproducibility of SWATH-MS. (a) a pair of whole replicates starting from the two aliquots of the twin trisomy sample (T1DS); **(b)** a pair of whole replicates starting from the two aliquots of the twin normal sample (T2N); **(c)** a pair of whole replicates starting from the two aliquots of a DS fibroblast sample from the unrelated invidiual; **(d)** a pair of whole replicates starting from the two aliquots of a normal fibroblast sample from an unrelated individual; **(e)** a pair of a temporal experiment replicates repeated for T1DS, run 1.5 months apart; **(f)** a pair of a temporal experiment replicates repeated for T2N, run 1.5 months apart; **(g)** Unsupervised hierarchical clustering analysis (HCA) of the final SWATH-MS data suggests the whole process replicates of the two aliquots always clustered together, suggesting that any data variance due to experimental and technical errors are far outweighed by the genetic and environmental differences between individuals. **(h)** The distribution of Pearson's correlation coefficient (R) between all possible binary experimental replicates between unrelated samples, twin samples, and temporally independent cell cultures. **(i)** Correlation between log2 FC of T1DS/T2N, the outcome of relative LFQ, between two independent cell cultures.

Supplementary Figure 4.

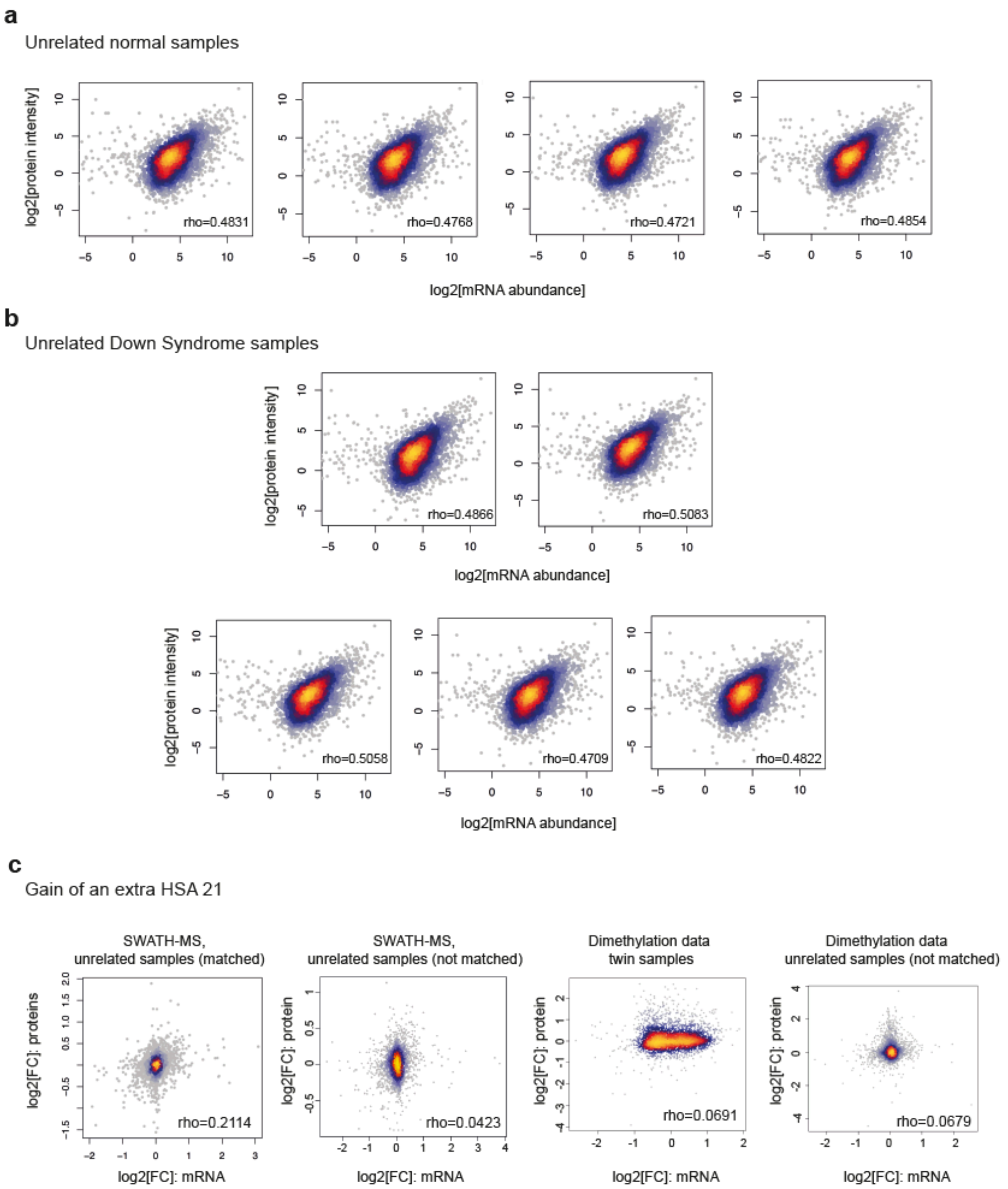

Supplementary Figure 4 mRNA-protein correlation analysis for unrelated samples with both mRNA and protein data. (a) Absoulte mRNA-protein correlation in Unrelated normal (N) samples. **(b)** Absoulte mRNA-protein correlation unrelated DS samples. **(c)** The log2 correlation between mRNA and protein T21/N FCs. Note that panels from left to right represent (1) SWATH data from 9 samples from (A) and (B), i.e., the same 9 samples measured by mRNA and proteins matched to each other, (2) SWATH data using averaged FC of T21 and Normal group where samples were not matched; (3) Dimethyl labeling shotgun proteomic data of twin samples correlated with mRNA, and (4) Dimethyl labeling shotgun data of FC of DS and Normal from the measurement of mixed all unrelated samples in each group (thus “unmatched”). Note that all four panels suggest while abundances correlated reasonably, transcript and protein FCs correlated weakly in both twin and unrelated samples.

Supplementary Figure 5.

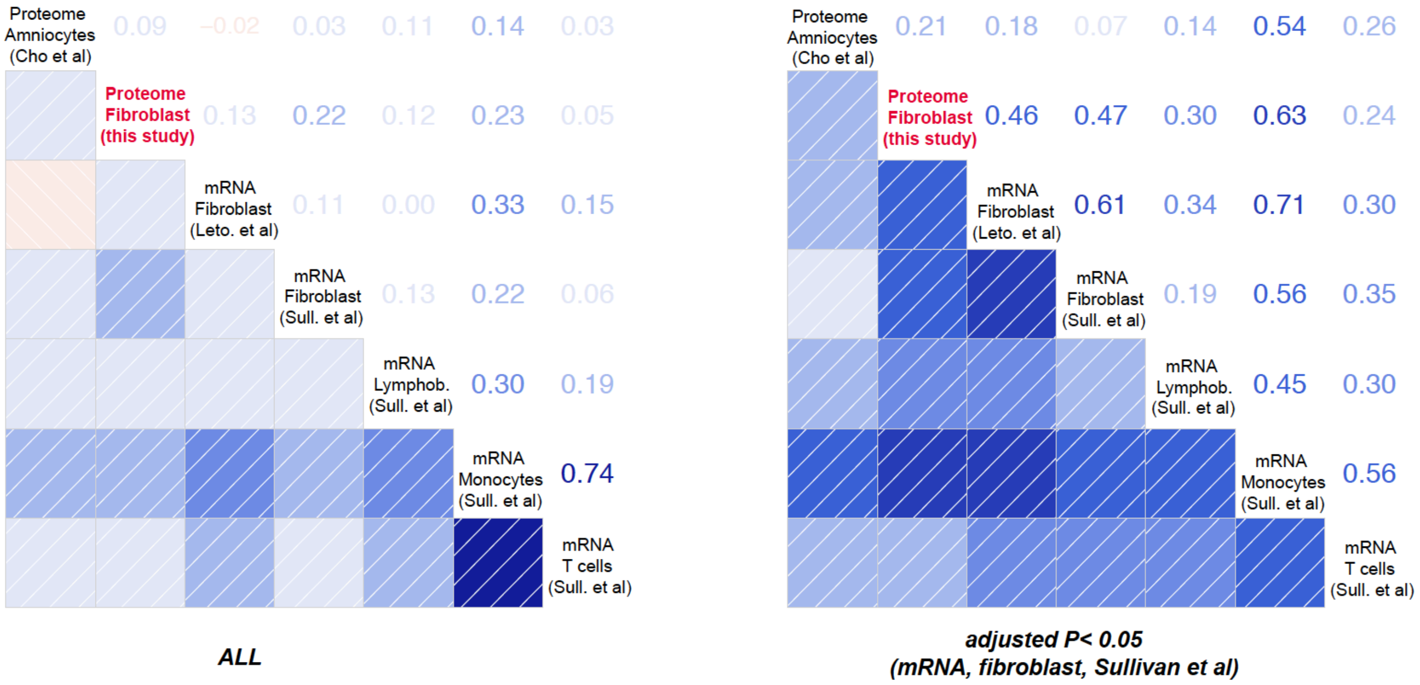

Supplementary Figure 5 Correlation analysis between proteomic and transcriptomic data sets for T21. (a) Genome-wide correlation. For proteomics, we use an old data set based on shotgun MS1 level quantification of T21/N for DS amniocytes (Cho et al 2013) which is the only one available to the best of our knowledge. For mRNA data, we use Sullivan et al which includes multiple cell types analyzed by the same group. Note Trisomy/Normal log2 Fold change (FC) of mRNA data only had weak correlation between different cell types. **(b)** Conservation of significantly transcriptional regulation using the list from the fibroblast transcriptomic data set (protein translated) of Sullivan et al. Note the significant transcriptional regulations in fibroblast cells are largely conserved between different cell types, and also have a bigger impact on the proteome. Interestingly, 28 out of 69 differential transcripts (about 40.5%) are coded from Chr21. Note even for the significantly regulated transcripts, the proteome data of aminocytes had a much lower correlation to all the other mRNA data set in other cell types, suggesting the in vitro culturing condition or experimental issues may lead to physiological alterations (in vitro cells were reported to stopped thrive after 8 generations). We therefore did not use this old proteomic data to confirm our SWATH-MS profiling on other observations in the present study.

Supplementary Figure 6.

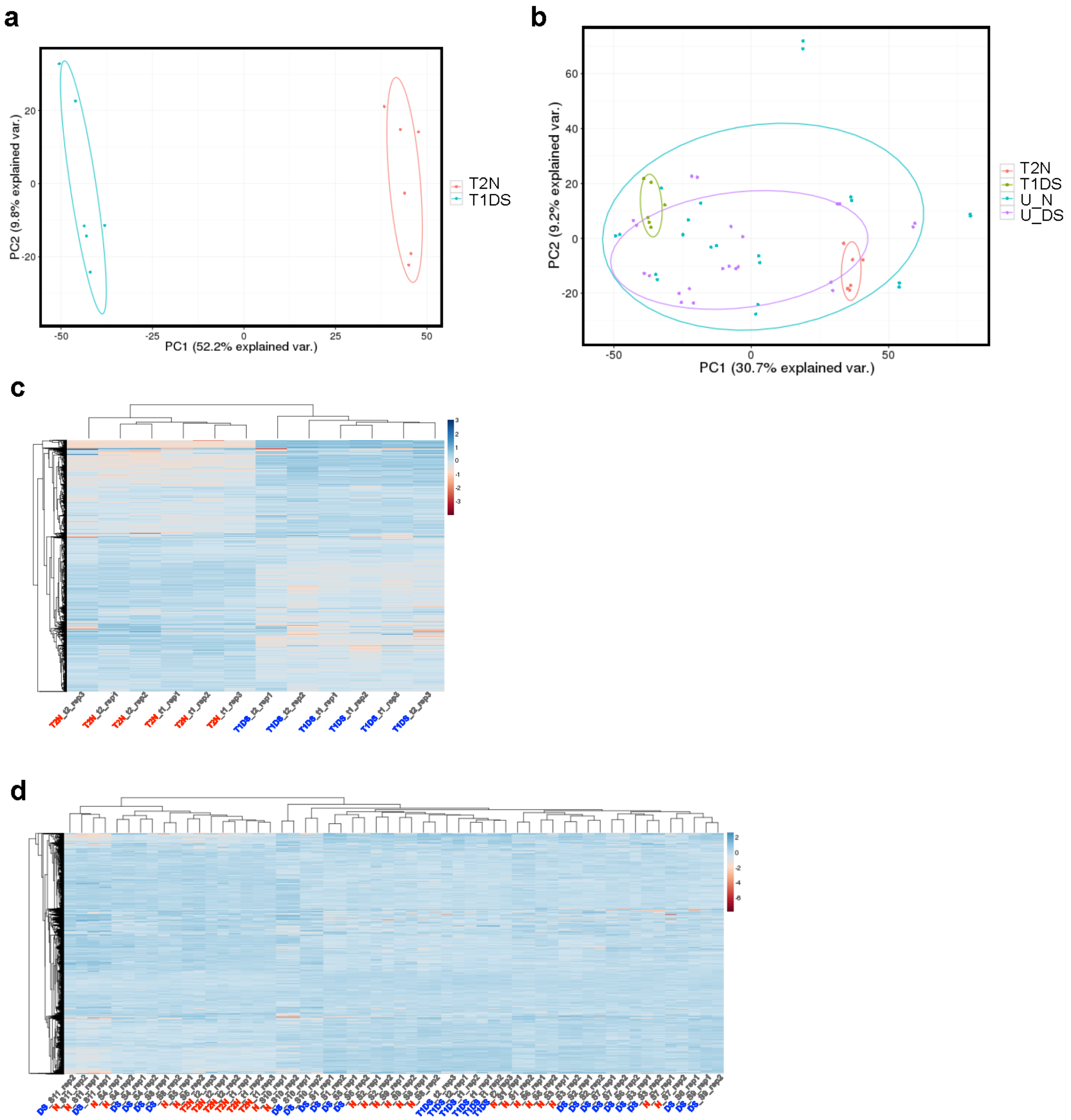

Supplementary Figure 6. Hierarchical clustering analysis (HCA) and principal component analysis (PCA) of twin samples and unrelated samples uncovers the significant individual variation of the proteomes from unrelated samples. To fairly position the samples from both twin samples and unrelated samples in HCA and PCA analyses, we firstly subtracted the mean value of each experimental batch to reduce the batch effects. **(a)** HCA of the twin samples. **(b)** HCA of the twin samples together with unrelated samples. **(c)** PCA of the twin samples. **(d)** PCA of the twin samples together with unrelated samples (the doublets of points denote the two experimental replicates of the same sample). Altogether, these results suggest by directly analyzing the fibroblast cells from the unrelated samples, the proteomic consequences of T21 cannot be revealed clearly due to the individual genomic variability and the huge biological variation (e.g., gender and age difference in Table S1). Thus, these results further highlight our approach of having the twin samples to firstly assess the biological difference without genetic variance and then to assess such differences from unrelated samples to explain the variability of DS phenotypes among humans.

Supplementary Figure 7.

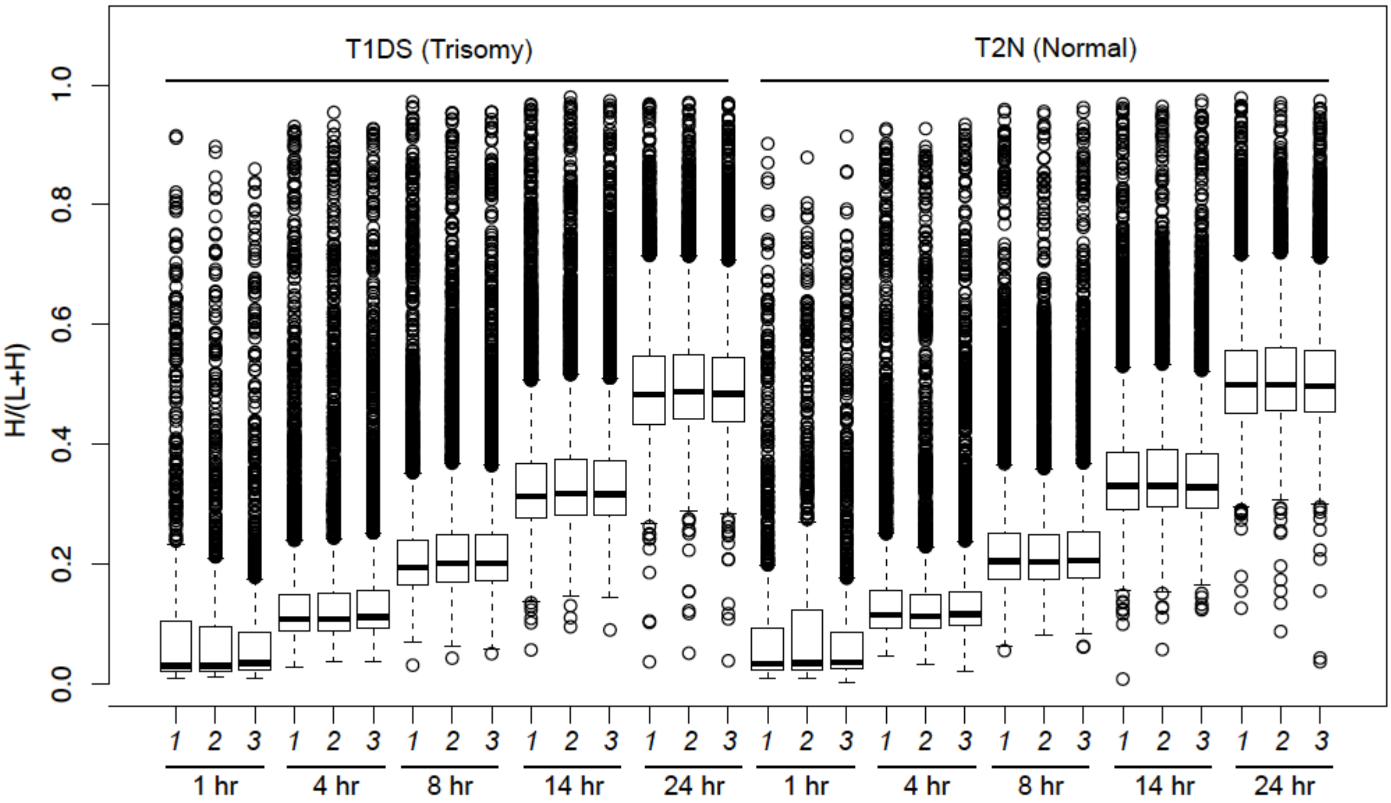

Supplementary Figure 7. Heavy amino acid incorporation in pSILAC experiment during 24 hours. . In a pSILAC design, researchers rely on the incorporation speed of the heavy isotopically labeled amino acids (such as K and R) into the cellular proteome to quantify protein turnover rate. Therefore, there is no need to achieve the full labeling in such a experiment. Here, we analyzed the two biological replicates during the labeling process up to 24 hours using LTQ orbitrap Elite and Maxquant quantification. We found the labeling percentages of H/(L+H) to be 50.08%, 50.76%, and 50.44% for the three biological replicates of T1DS cells and comparably, 51.61%, 51.95%, and 51.72% for T2N cells.

Supplementary Figure 8.

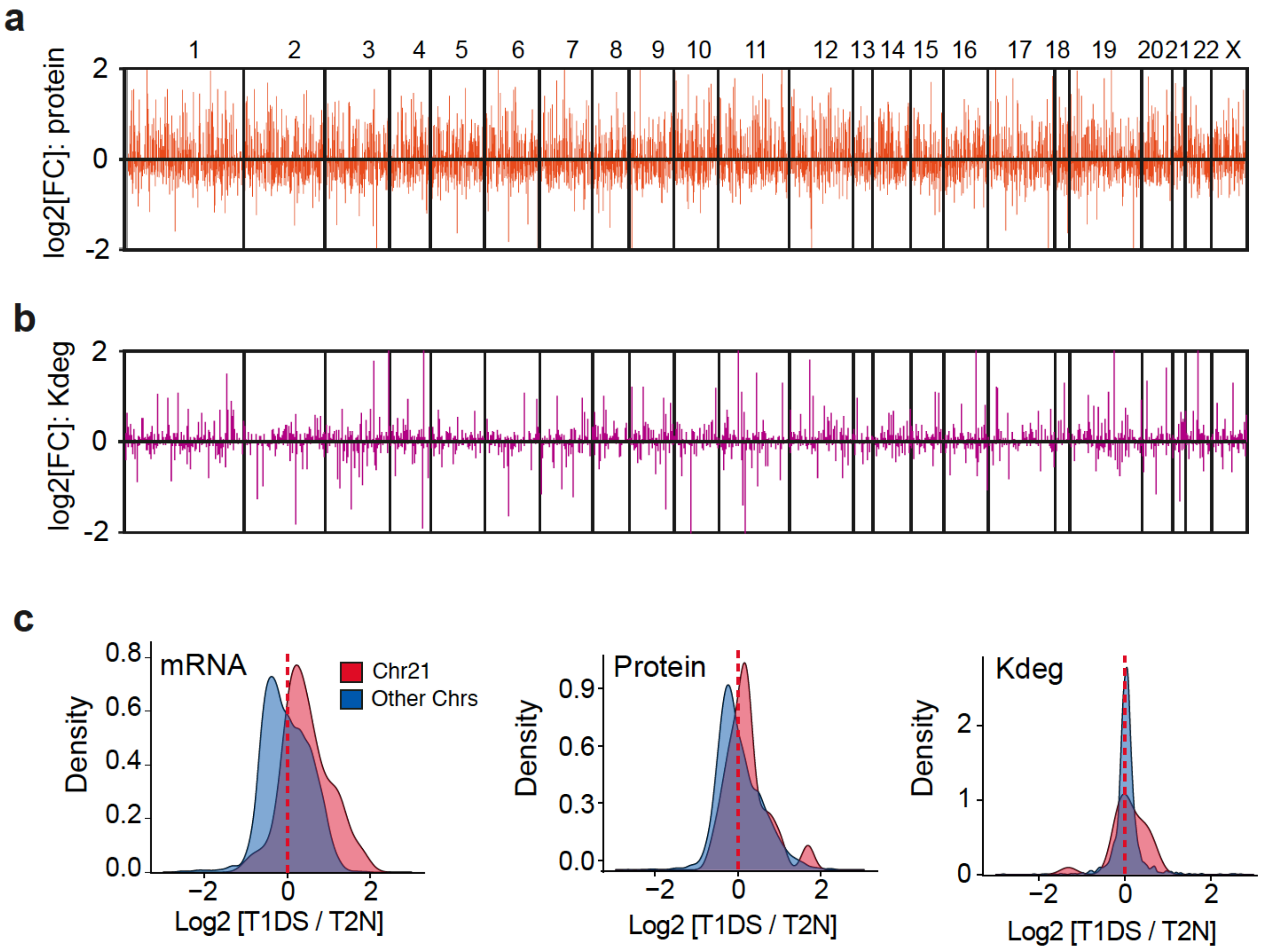

Supplementary Figure 8 Overall regulations at protein and protein degradation levels in T1DS/T2N comparasion. (a-b) The T1DS/T2N FC distributions at mRNA, protein, and protein degradation levels along the chromosomal locations. **(c)** The fold-change distributions of T1DS/T2N for HSA21 coded proteins and proteins coded by other chromosomes for mRNA, protein, Kdeg levels. The T1DS/T2N FC of HSA21 mRNAs, proteins and protein degradation are 1.38, 1.13 and 1.07 respectively, all higher than that for genes encoded by other chromosomes. mRNA data was downloaded from the publication of Letourneau et al where the same twin samples were analyzed using RNA sequencing.

Supplementary Figure 9.

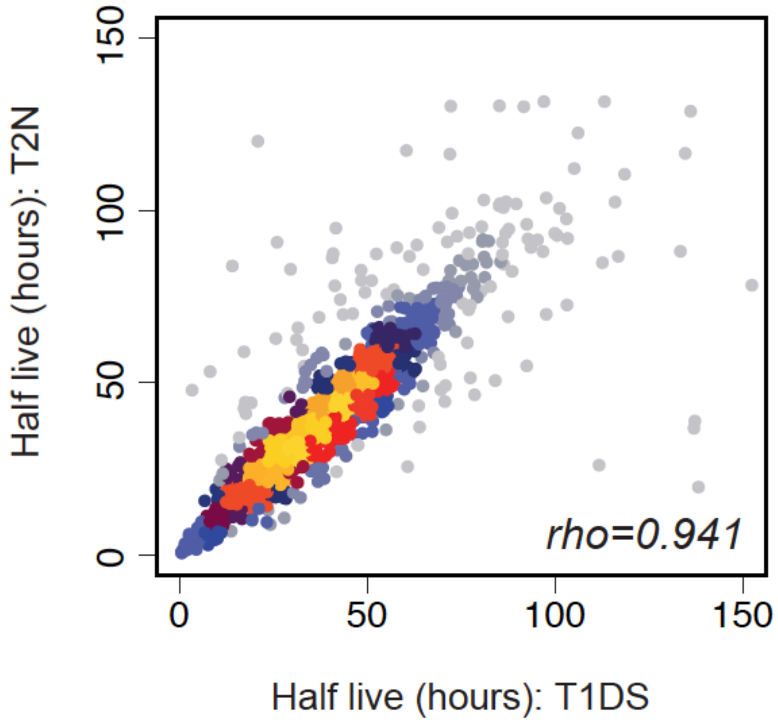

Supplementary Figure 9. Correlation between determined protein half-lives of T1DS and T2N cells. The degradation rate could be converted into protein half-lives. Most proteins (>98.5%) have a half-life less than 100 hours for both T1DS and T2N, with a high correlation between the two conditions. The median half-lives is estimated to be 37.17 hours and 38.92 hours for T1DS and T2N fibroblasts in our data**.**

Supplementary Figure 10.

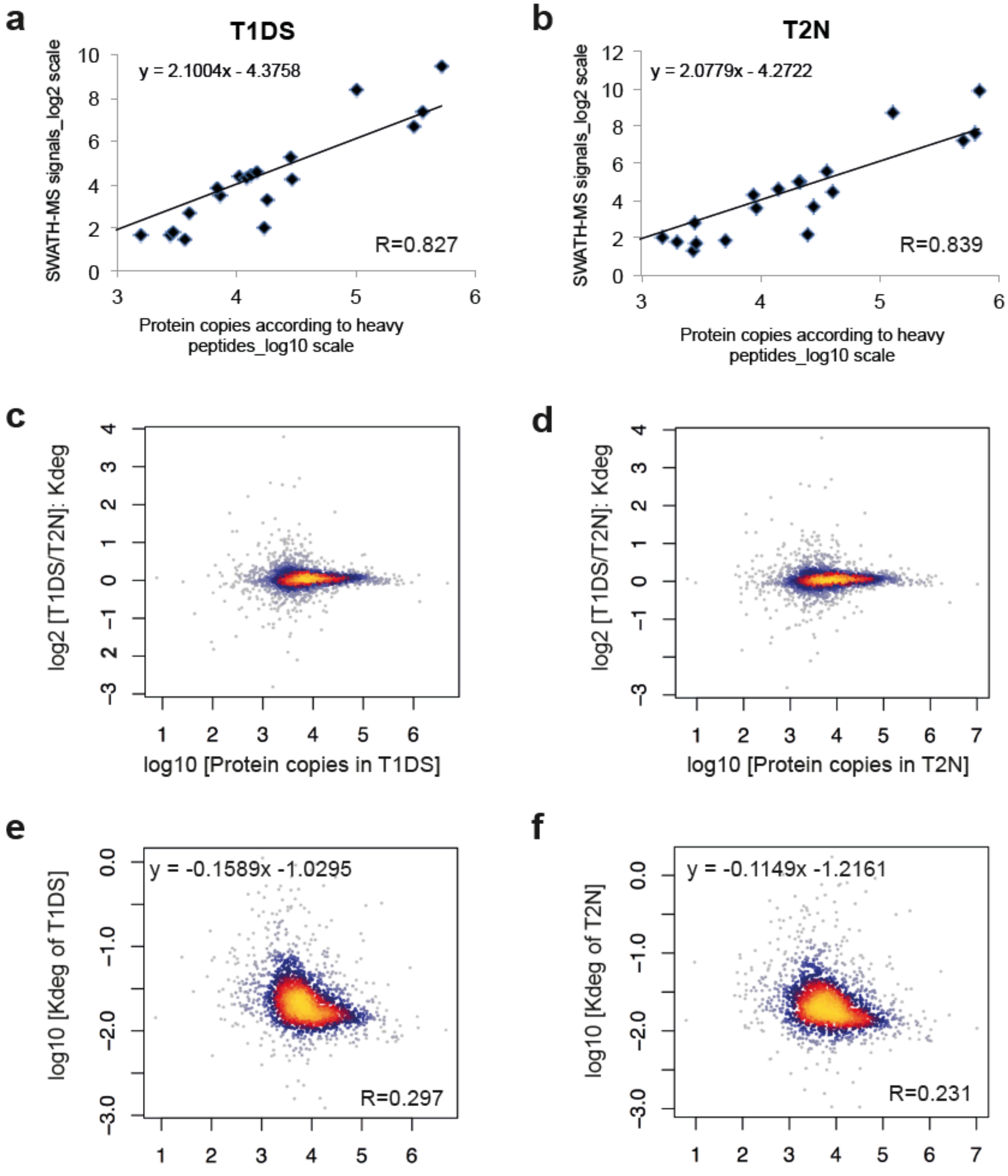

Supplementary Figure 10. Determination absolute protein copies per cell and its relationship to protein degradation rate. (a-b) The linear correlation between the SWATH-MS intensities of 19 anchor proteins and the protein copies measured by the heavy peptides in T1DS and T2N samples, severing as the foundation for estimating absolute protein copies per cell by aLFQ. **(c-d)** High abundant proteins tend to have less variation (regulation) of *Kdeg* between T1DS and T2N. **(e-f)** Consistent to (c-d), the absolute *Kdeg* values are smaller for high abundant proteins in both T1DS and T2N, suggesting the high abundant proteins carrying house keeping functions tended to have small turnover rates.

Supplementary Figure 11.

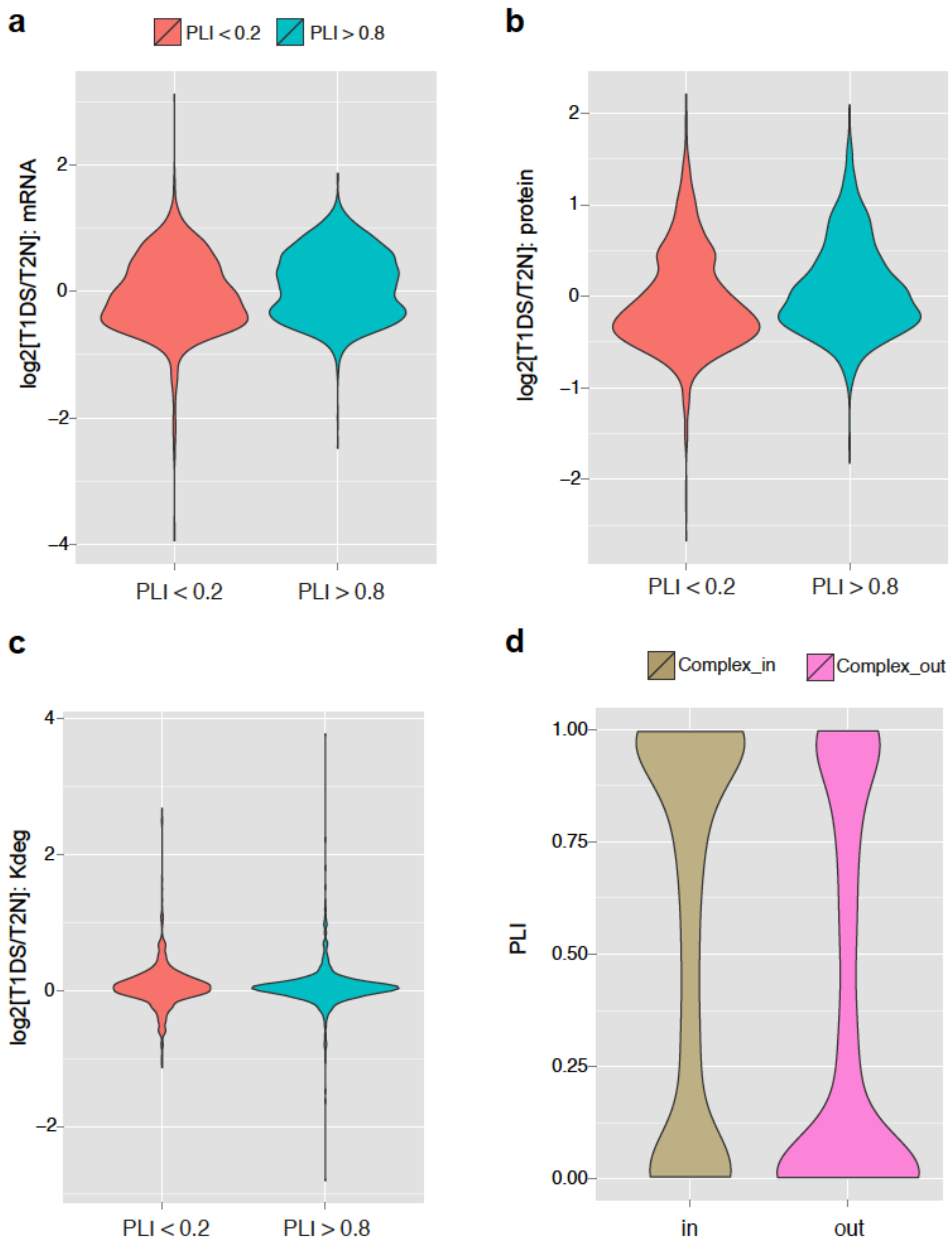

Supplementary Figure 11. Relationship between mRNA, protein and *Kdeg* regulations and gene haploinsufficiency score (PLI score) (*67*). A fraction of genes in the human genome are categorized as haploinsufficient (*67*) since the loss-of-function variants of one allele results in a recognizable phenotype. **(a-b)** The FC of mRNA and proteins between T1DS and T2N is slightly higher for haploinsufficenct genes, probably because upregulation of protein functions of haploinsufficient genes are important for stress induced by T21. **(c)** The proteins of high haploinsufficiency (i.e. intolerant to loss-of-function variants, PLI > 0.8) are less significantly regulated (flat distribution of the teal violin) and slightly less degraded compared to other proteins. **(d)** The proteins participating in protein complexes as subunits have a higher haploinsufficiency score (as compared to complex_out proteins), suggesting their protein dosage is more important to stabilize the phenotype (as compared to complex_out proteins).

Supplementary Figure 12.

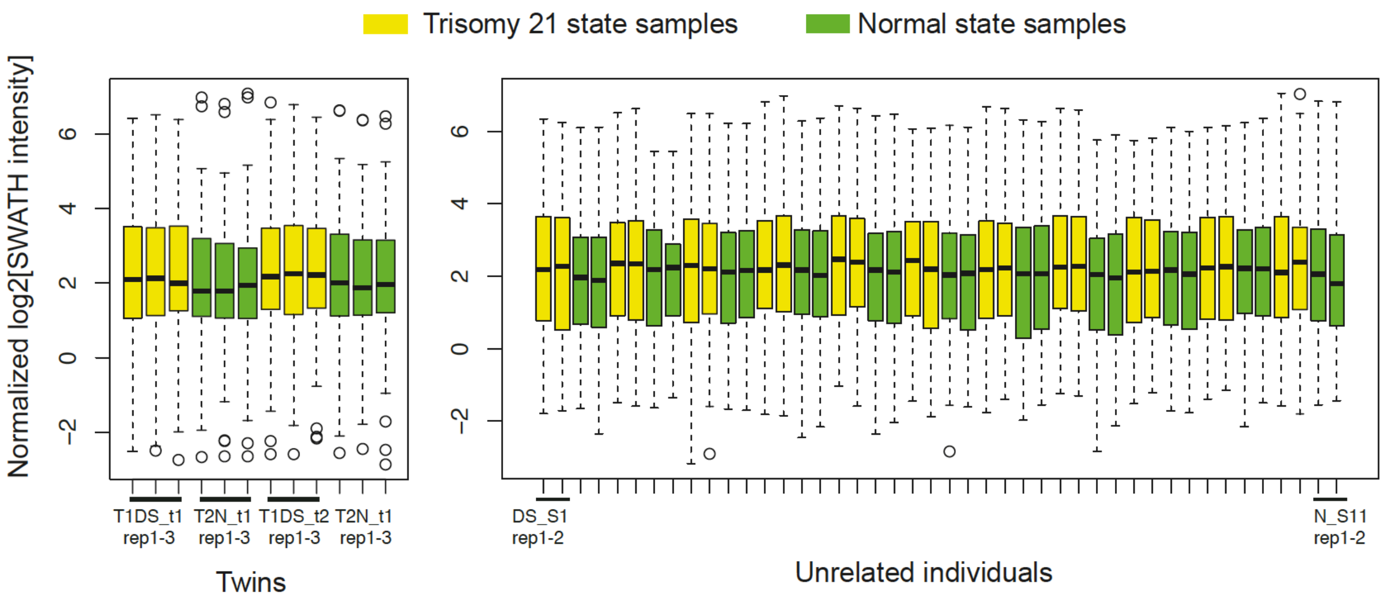

Supplementary Figure 12. Higher expression of HSA21 proteins in all T21 state samples compared to Normal controls. The SWATH-MS intensities for the HSA21 proteins distributed in all the 56 samples measured. Yellow and green boxes denote the trisomy and normal states in twin samples (left panel) and genetically unrelated samples (right panel). As expected these HSA21 proteins displayed on average a higher expression in all T21 cases compared to controls, a primary proteomic feature of T21 cells.

Supplementary Figure 13.

a

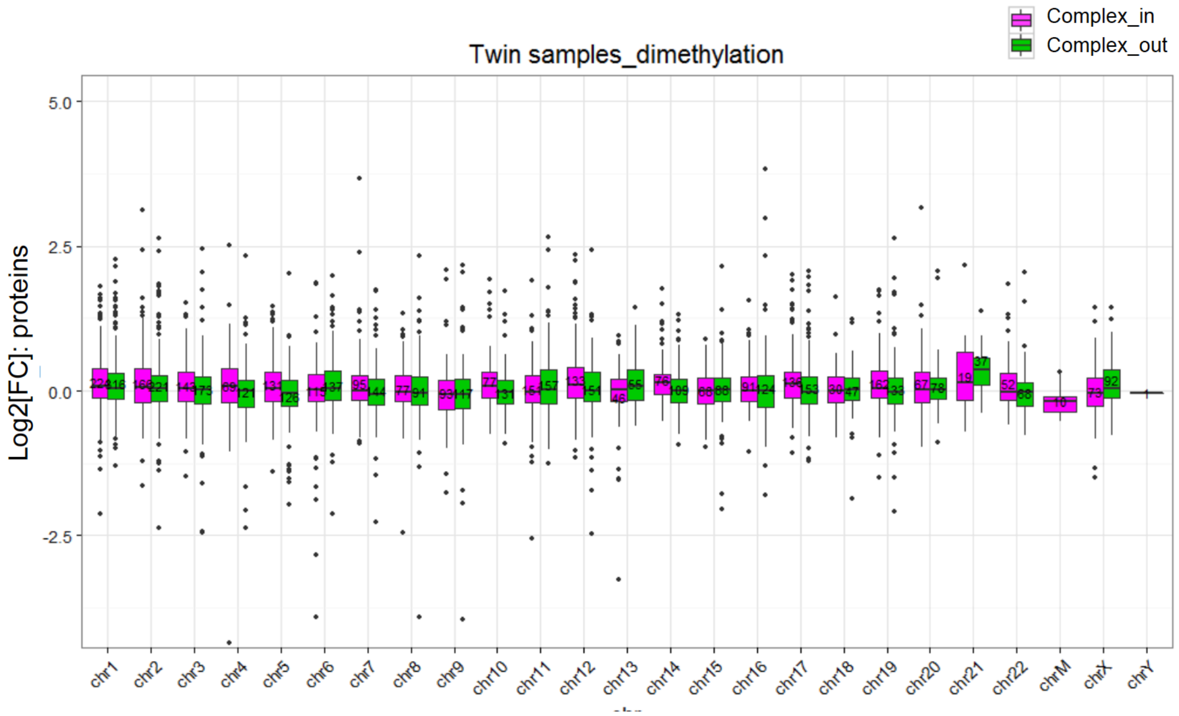

b

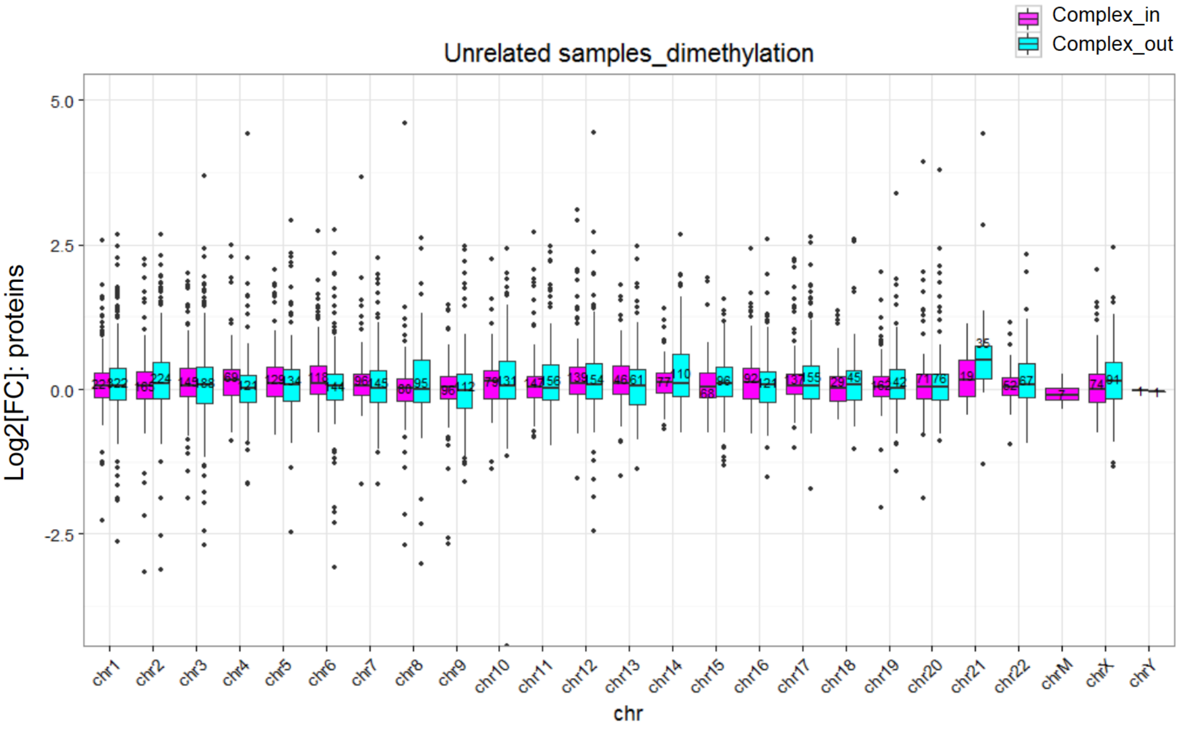

Supplementary Figure 13. Confirmation of the protein complex stoichiometry based buffering mechanism against T21-induced CNVs by the independent data set of dimethyl labeling shotgun proteomics. Dimethyl labeling shotgun proteomic data for twin sample mixtures distributed across all the chromosomes. The number of proteins quantified on each chromosome is noted. **(b)** Dimethyl labeling shotgun proteomic data for unrelated sample mixtures distributed across all the chromosomes. Protein T21/N FCs of the "complex_in" group were lower than "complex_out" group for both sample sets (P<0.05), consistent to **Fig. 3c**.

Supplementary Figure 14.

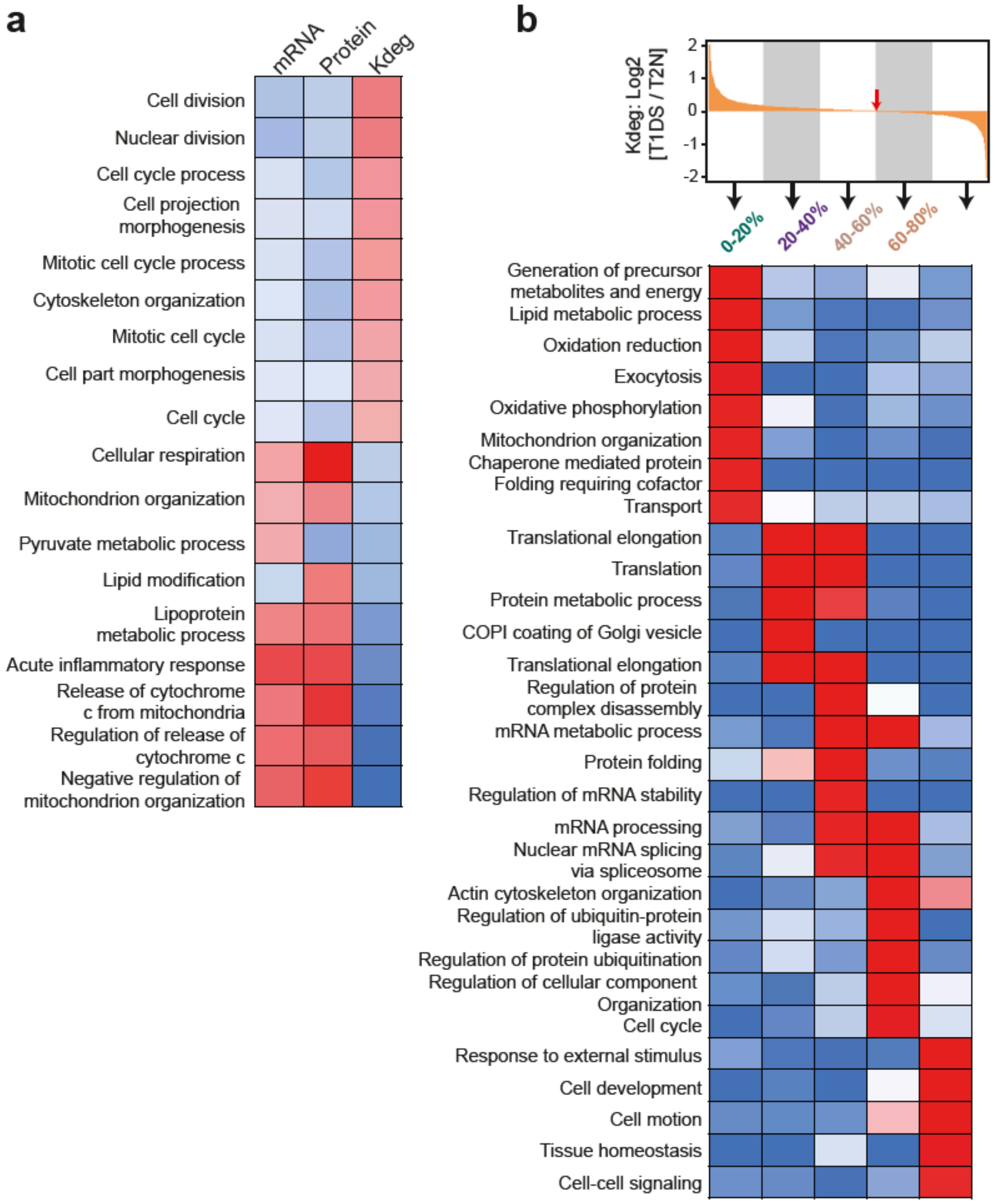

Supplementary Figure 14. Expanded lists illustrating all significant biological processes due to T21. (a) Significant, overlapping biological processes after GSEA analysis at mRNA, protein, and *Kdeg* levels. The LOR ratios of T1DS/ were visualized on a red/blue color scale, with blue color denoting LOR>0 and red denoting LOR<0 (See Methods and Materials). **(b)** The ranked list of protein degradation regulation under T21 stress was divided into five segments of proteins, which were analyzed for the GO biological processes enriched in each segment.

Supplementary Figure 15.

a

	SWATH_1895 annotated (with Kdeg quantified)	SWATH_3199 annotated	Dimethy-_4009 annotated
Cytosol	0.380	0.280005727	0.158
Mitochondrion	-0.156	-0.192811831	-0.165
Nucelolus	-0.015	-0.06792711	-0.051
Spliceosome	-0.149	-0.13577255	-0.021
Cytosolic ribosome	0.307	0.30395875	0.369
ER	-0.194	-0.213920914	-0.097
Proteasome	0.236	0.235708253	0.154
Lysosome	-0.099	-0.129084698	-0.014
Golgi	0.013	-0.017042071	0.022
Chromosome	0.170	0.085785597	0.055
Peroxisome	-0.162	-0.175840156	-0.099
Cell Surface	-0.110	-0.144400888	-0.055
Extracellular matrix	0.027	-0.119948498	-0.030
Plasma membrane	0.071	0.02151949	0.065

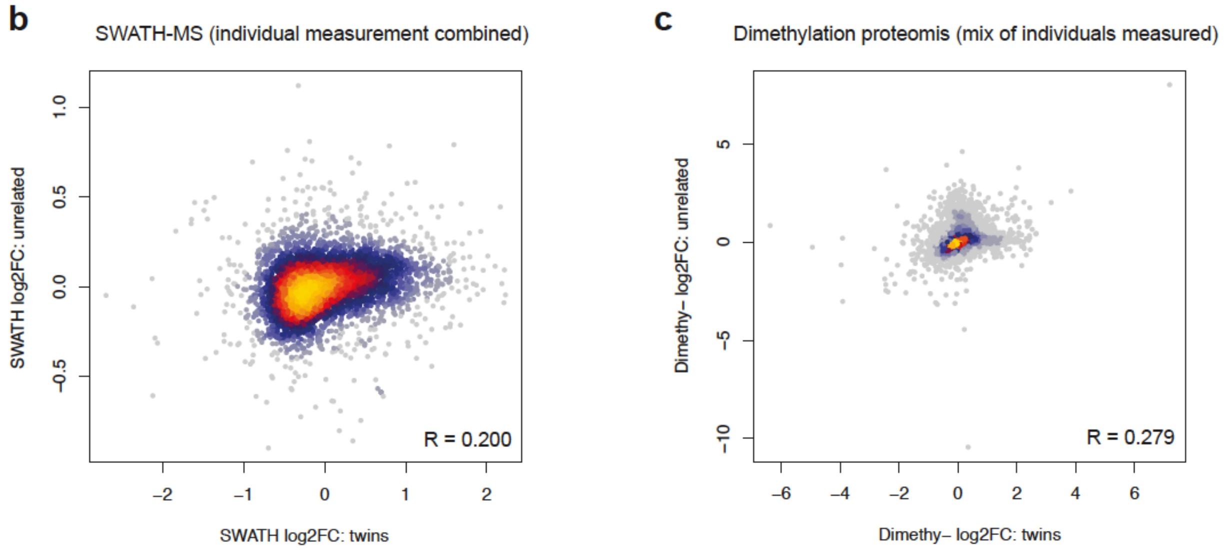

Supplementary Figure 15. The consistency of regulated proteome between twin and unrelated sample sets. (a) Similar conclusions of regulation of each organelle proteome can be made by SWATH-MS data (with Kdeg quantified or not) and the independent dimethyl labeling-shotgun proteomic measurement. The numbers denote protein numbers included in the cellular component annotation. **(b)** Modest correlation of T21/Normal between twin samples and unrelated samples quantified by SWATH-MS. **(c)** Modest correlation of T21/Normal between twin samples and unrelated samples quantified by dimethyl labeling-shotgun proteomics. Note for dimethyl labeling, the unrelated samples were firstly mixed into either T21 or Normal group and then labeled with dimethyl labeling reagent (see Method), which could provide a better estimate on differential proteome between groups, but failed to address inter-individual variability compared to SWATH-MS label free quantification.

Supplementary Figure 16.

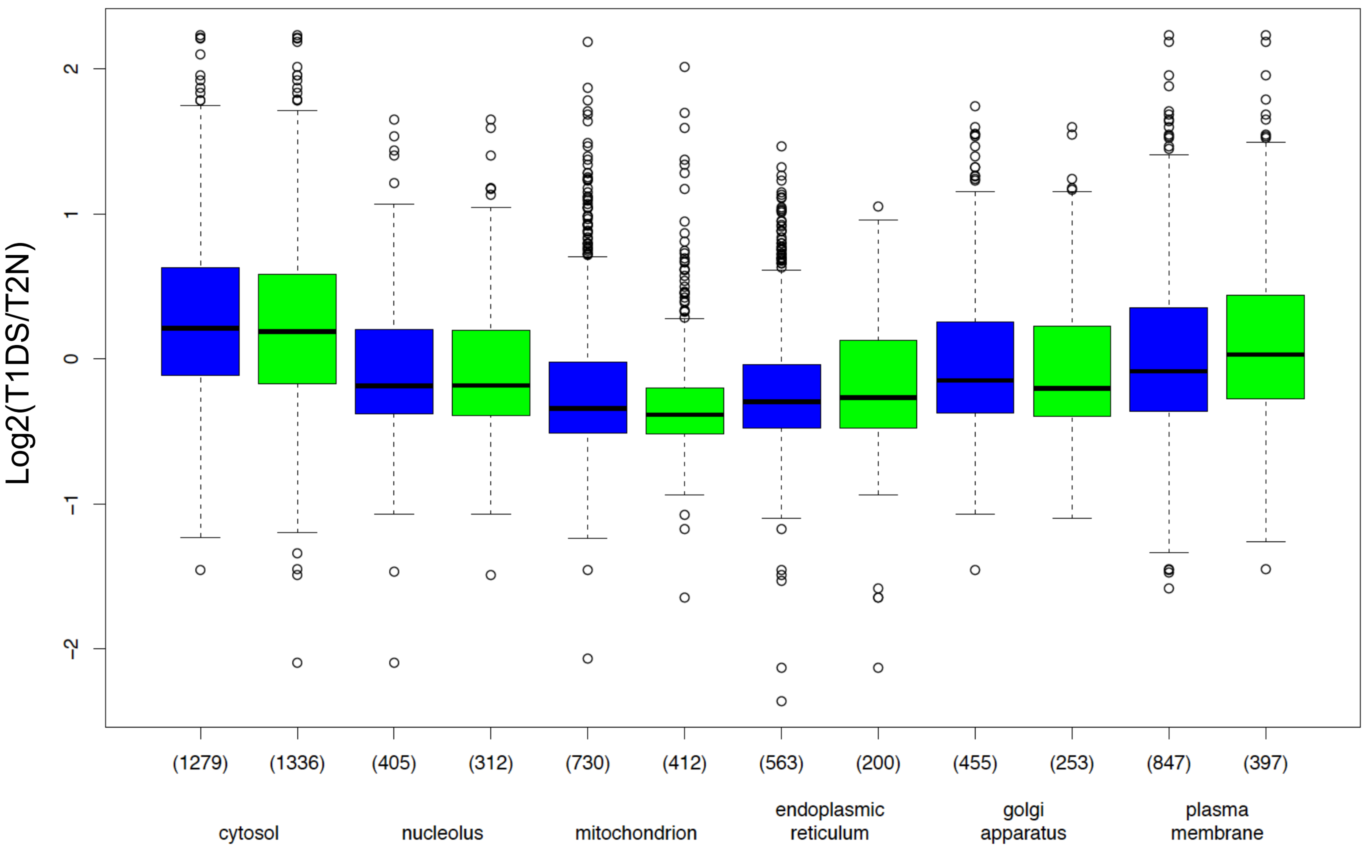

Supplementary Figure 16. Organelle specific regulation is not dependent on the annotation database. Here a recently published subcellular map of the human proteome derived from antibody-based microscopy experiments (Thul et al), was used as as an alternative annotation method of organelles. Blue boxes denote the BioMart annotation used in the present study whereas green boxes denote classification using results of Thul et al. The conclusion of organelle proteome regulations of log2(T1DS/T2N) can be derived from both classification systems.

Supplementary Figure 17.

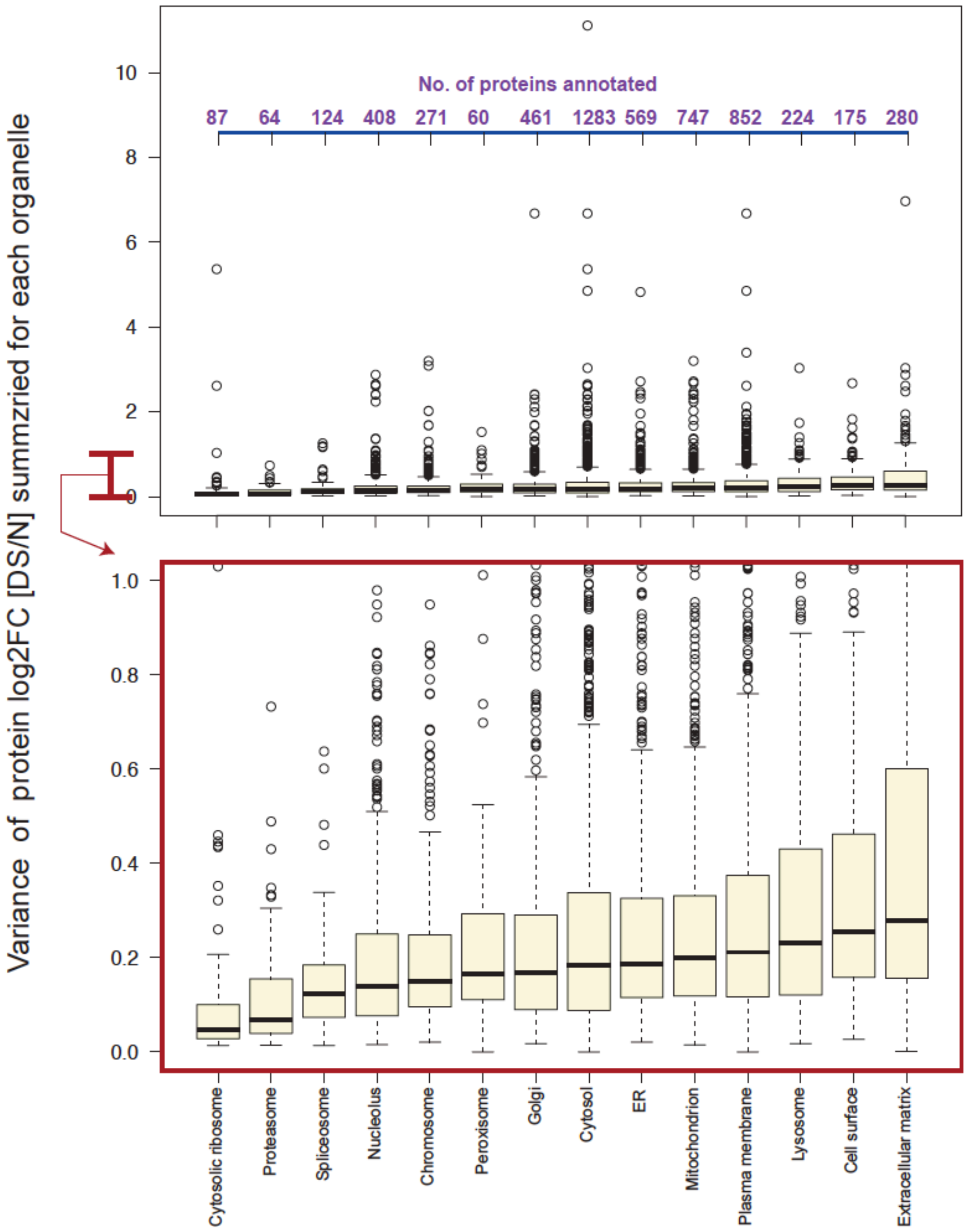

Supplementary Figure 16. The variance of the protein log2FC of DS versus N according to unrelated samples. Note the variance of proteins in each organelle was distributed as boxplots, and the lower panel is a zoomed in visualization of the upper panel, describing the organelle proteomic variation. Note the variance here, as discussed, should include both individual genomic variability and DS effects.

Supplementary Figure 18.

Supplementary Figure 18.

a. Twin samples (T1DS and T2N).

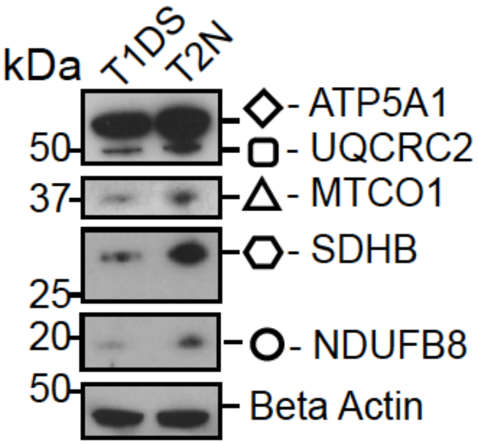

b. 11 pairs of unrelated samples.

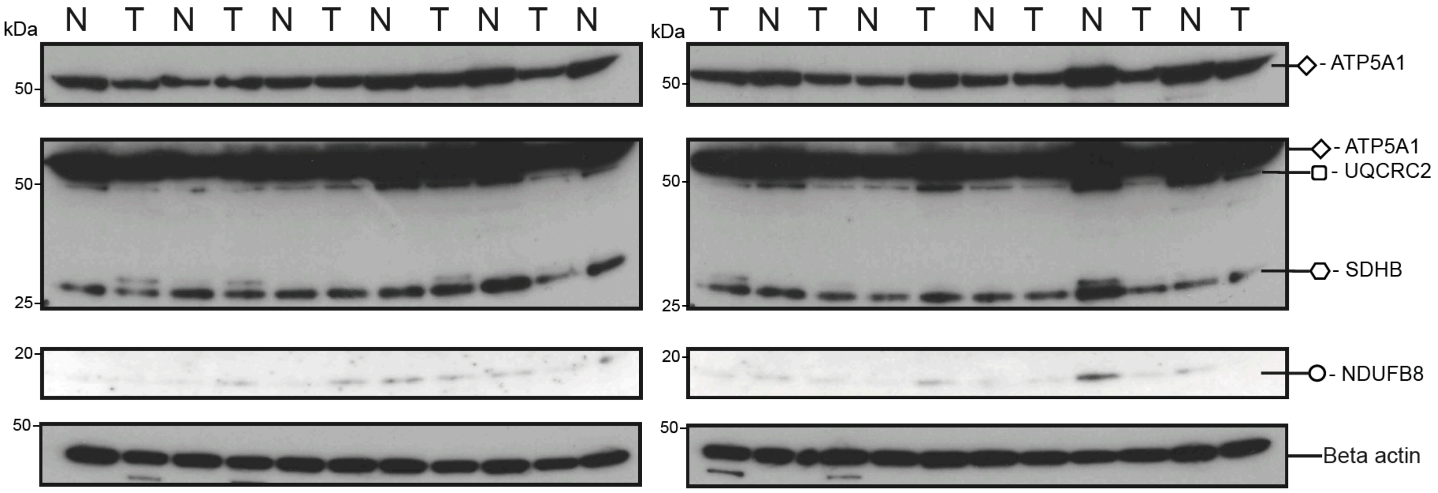

Supplementary Figure 18. Western blotting verification of the mitochondrial proteome changes upon T21. Note that the significance of downregulation of mitochondrial proteins is higher for twin samples **(a)** but lower for unrelated samples **(b)** due to the individual variability among unrelated samples. This is the consistent to our SWATH-MS data (e.g., **Fig. 6** in the main text).

Supplementary Figure 19.

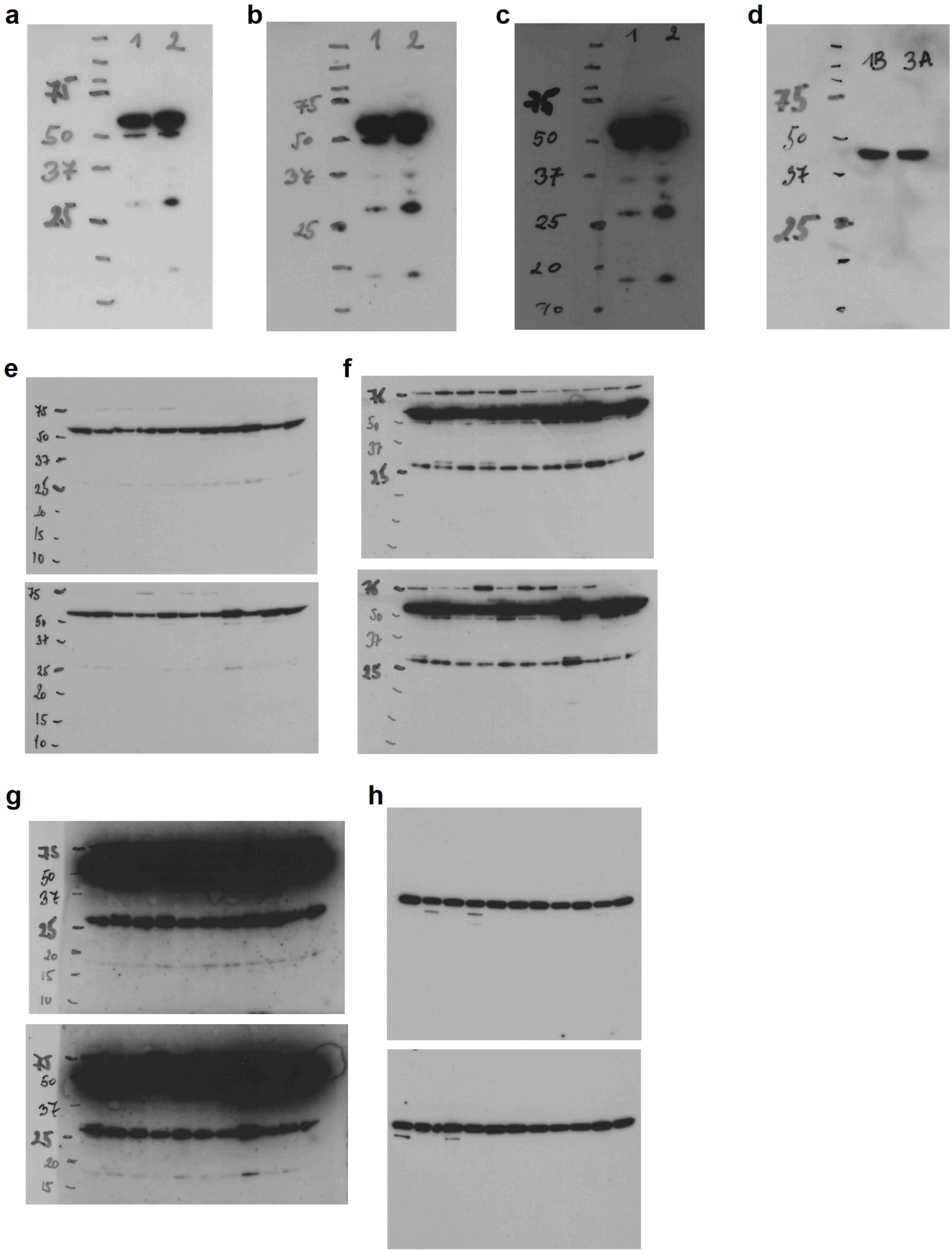

Supplementary Figure 19 Full membranes detected for Western blots shown in the main and supplementary figures. An antibody cocktail contains Anti-NDUFB8 (~20KD), Anti-SDHB (~30KD), Anti-UQCRC2 (~48KD), Anti-MTCO1 (~40KD), Anti-ATP5A1 (~55KD) was used. With this antibody cocktail and different length of exposure time from 2 min to about 0.5-1 hour (**a-b-c** for the twin samples, **e-f-g** for the unrelated samples) different antibodies generated optimal bands that were cut for the figures. The panel **d** and **h** denote corresponding Anti-actin bands as controls.

Supplementary Table

Supplementary Table 1. Summary of previous literatures supporting the usage of fibroblast cells to study DS.

Author	Journal	Year	Cells used	Phenotypes	Insights and conclusions	PMID
**Kimura et al**	Free Radical Biology & Medicine	2005	Skin fibroblast cells	Imbalance of SOD activity and telomere repeat loss for accelerated aging	Down syndrome cells had a significantly slower proliferative rate, but attain replicative senescence at similar population doubling.	15993336
**Prandini et al**	The American Journal of Human Genetics	2007	Lymphoblastoid and Fibroblast Cells	Extensive phenotypic variability	Only 39% and 62% of Chr21 genes in lymphoblastoid and fibroblast cells, respectively, showed a significant difference between DS and normal samples, although the average up-regulation of Chr21 genes was close to the expected 1.5-fold in both cells.	17668376
**Cataldo et al**	The American Journal of Pathology	2008	Forearm skin fibroblasts	Alzheimer-Related Endosome Pathology	Like neurons in both AD and DS brains, DS fibroblasts exhibit increased endocytic uptake, fusion, and recycling, and trafficking of lysosomal hydrolases; The DS fibroblast model is likely to prove valuable in further dissecting alterations in function of the endosomal- autophagic-lysosomal system	18535180
**Jiang et al**	Proc Natl Acad Sci U S A.	2010	Forearm skin fibroblasts	Morphological and functional endocytic abnormalities	Alzheimer's-related endosome dysfunction in Down syndrome is Aβ-independent but requires APP	20080541
**Dogliotti et al**	Toxicology in Vitro	2010	Skin fibroblasts	Apoptotic response of DS fibroblasts to Okadaic acid	DS Fibroblasts have a baseline of apoptosis higher than normal fibroblasts and are more susceptible to the pro-apoptotic effect of Okadaic acid.	20006980
**Micali et al**	Nucleic Acids Research	2010	Skin fibroblasts	Apoptotic phenotype	Prep1 of Chr 21 increases the sensitivity of cells to genotoxic stress. The differences in Prep1 level can have drastic effects on apoptotic phenotype.	20110257
**Valenti et al**	Biochemical Journal	2011	Foetal skin fibroblasts	Mitochondrial dysfunction contributing to DS pathogenesis	Deficit of complex I activity in human skin fibroblasts with chromosome 21 trisomy and overproduction of reactive oxygen species by mitochondria	21338338
**Gimeno et al**	*Biochimica et Biophysica Acta*	2013	Primary skin fibroblasts	Progeroid-like phenotype;Decreased cell proliferation and higher oxidative stress	High oxidative stress in DS is a very early development event in fetus fibroblasts	24184606
**Piccoli et al**	Human Molecular Genetics	2013	Primary skin fibroblasts	Impairment of mitochondrial function	Chronic pro-oxidative state and mitochondrial dysfunctions are more pronounced in fibroblasts from Down syndrome foeti with congenital heart defects	23257287
**Sullivan et al**	eLIFE	2016	Fibroblast and lymphoblastoid cell lines, circulating monocytes and T cells	Protective and deleterious immune effects	Transcriptome analysis revealed that trisomy 21 consistently activates the interferon transcriptional response in all the cell lines tested.	27472900

Note there are at least three phenotypes of DS potentially reflected by fibroblast cells: 1) Endosome Pathology/ functional endocytic abnormalities related to Alzheimer; 2) Apoptotic phenotype in DS; 3) Decreased cell proliferation, higher oxidative stress (impaired mitochondrial function), likely related to progeroid.
