# Supplementary material for: Systematic proteome and proteostasis profiling in human Trisomy 21 fibroblast cells
Source: Nat Commun. 2017 Oct 31;8:1212. doi: 10.1038/s41467-017-01422-6 (PMC5663699; doi:10.1038/s41467-017-01422-6)
Supplement: Supplementary file 3 — Description of Additional Supplementary Files [file 41467_2017_1422_MOESM3_ESM.pdf]

Description of Additional Supplementary Files

File Name: Supplementary Data 1
Description: Sample information for the genetically unrelated individuals.

File Name: Supplementary Data 2
Description: Relative and absolute proteomic quantification by SWATH-MS.

File Name: Supplementary Data 3
Description: T21 associated RNA, protein, and protein degradation regulations.

File Name: Supplementary Data 4
Description: The Reactome processes significantly regulated due to T21 in twin samples and unrelated samples.

File Name: Supplementary Data 5
Description: Significant, overlapping biological processes after GESA analysis at mRNA, protein, and Kdeg levels.

File Name: Supplementary Data 6
Description: Enrichment analysis of GO biological processes for all the five segments evenly divided based on the T1DS/T2N fold-change of protein degradation.

File Name: Supplementary Data 7
Description: Enrichment analysis of GO cellular component for all the five segments evenly divided based on the T1DS/T2N fold-change of protein degradation.
